# Supplementary material for: A Study on Potential Sources of Perineuronal Net-Associated Sema3A in Cerebellar Nuclei Reveals Toxicity of Non-Invasive AAV-Mediated Cre Expression in the Central Nervous System
Source: Int J Mol Sci. 2025 Jan 19;26(2):819. doi: 10.3390/ijms26020819 (PMC11765860; doi:10.3390/ijms26020819)
Supplement: Supplementary file 1 [file ijms-26-00819-s001.zip › ijms-3409744-supplementary.pdf]

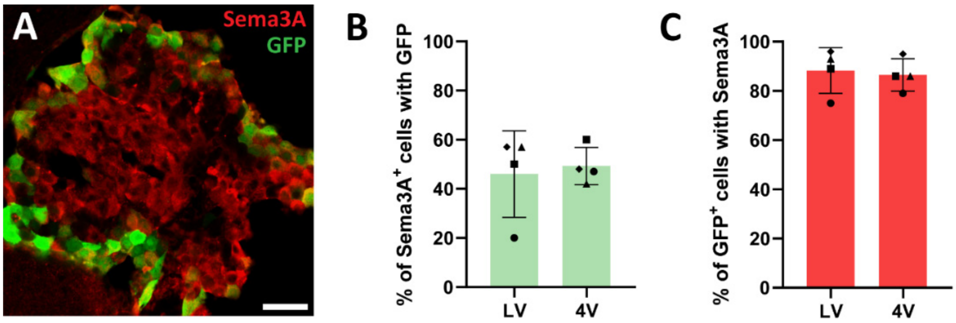

Supplementary Figure S1. Transduction efficiency of AAV1-CAG-GFP in the ChP. **(A)** GFP+ cells in the ChP following injection of AAV1-CAG-GFP. Sema3A+ cells are shown in red. **(B)** Around 50% of Sema3A+ cells in the ChP of the lateral ventricle (LV) and the IV ventricle (4V) express GFP. **(C)** Around 90% of GFP^{+} cells in the ChP of the lateral ventricle (LV) and the IV ventricle (4V) are Sema3A-positive (N = 4; each mouse is represented by a shape symbol). Scale bar: 40µm (A).

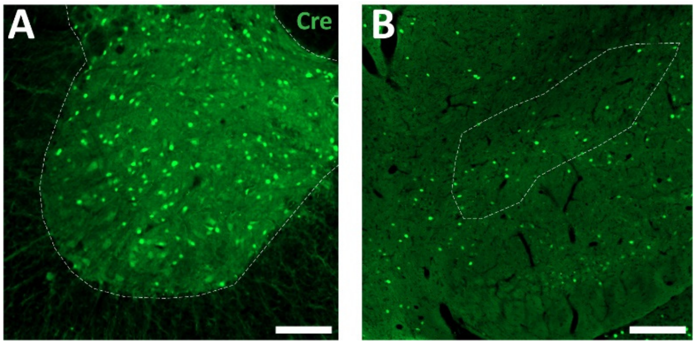

Supplementary Figure S2. Cre expression in the ventral horn of the SC and in the SNc of global KO mice. **(A)** Strong transduction of Cre in the ventral horn of the SC. **(B)** Scattered Cre^{+} cells in the SNc of global KO mice. Regions of interest are outlined by dashed lines. Scale bar: A: 150µm; B: 200µm.

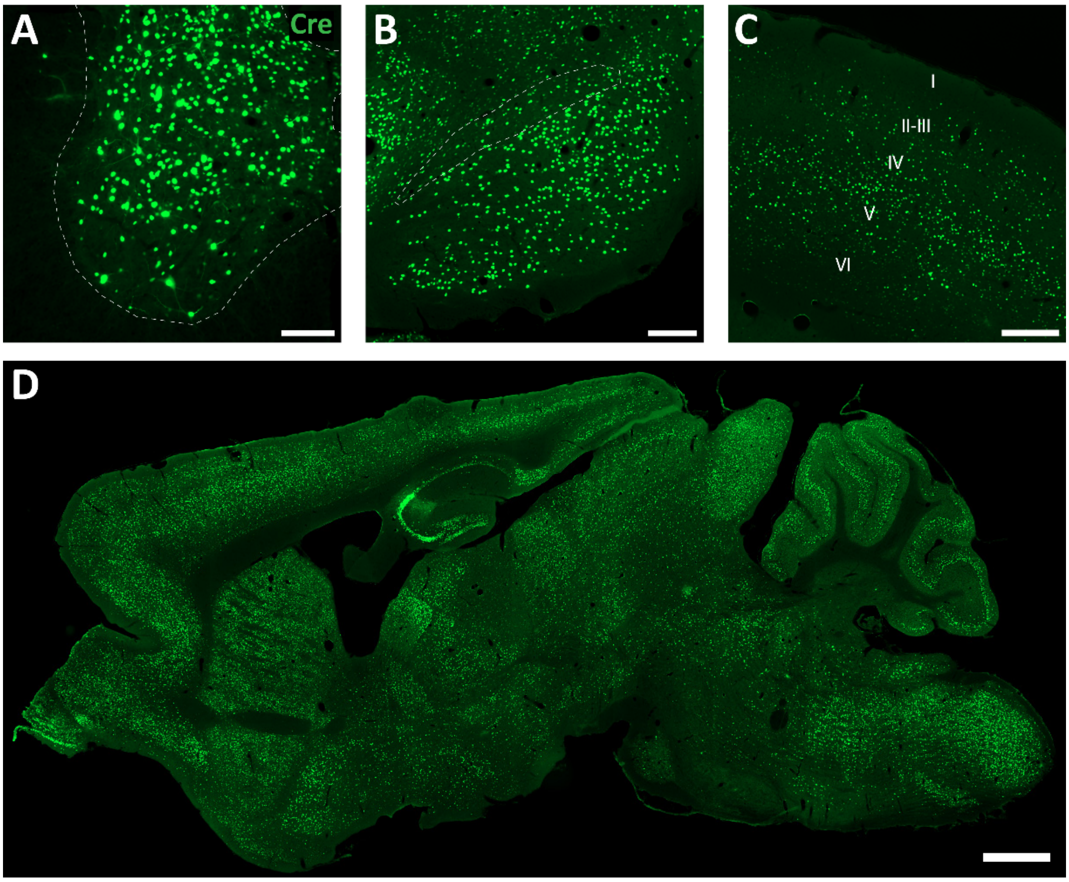

Supplementary Figure S3. Cre expression in the CNS of CD-1 Cre mice. **(A-D)** Strong expression of CreGFP in coronal sections containing the ventral horn of the SC (A; dashed lines), the SNc (B; dashed lines), the S1bf (C; cortical layers are indicated by roman numbers). (D) Sagittal section showing numerous CreGFP+ neurons throughout the brain. Scale bar: A: 150µm; B: 200µm; C: 300µm; D: 800µm.

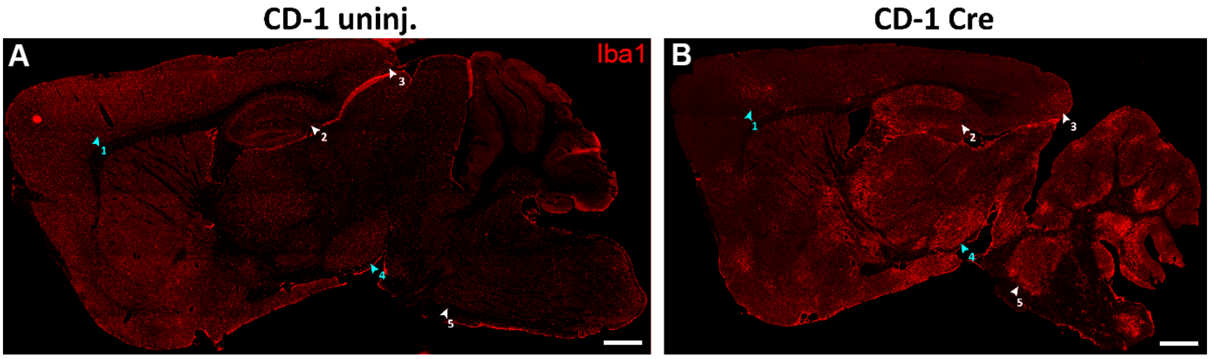

Supplementary Figure S4. Increased Iba1 expression in the brain of CD-1 Cre mice. **(A, B)** Strong microglia reactivity throughout the brain is observed in CD-1 Cre mice (B) when compared to CD-1 uninjected mice (A). Arrowheads in (B) point to areas of apparent microgliosis, including motor areas [e.g. primary motor cortex (cyan arrow 1), substantia nigra (cyan arrow 4)] and non-motor areas [e.g. hippocampus (white arrow 2), visual cortex (white arrow 3), principal sensory trigeminal nucleus (white arrow 5)]. Arrowheads in (A) point to the same areas of a control mouse. Scale bar: 800μm (A, B).

Antibodies	Supplier and catalogue number	Concentration	Species of origin	Characterisation	Controls
**anti-calbindin**	Swant, 300	1:1500	Mouse	By immunostaining on calbindin knock-out mice (absence of specific staining) and by western blot (it recognises a single band of 27-28 kDa in mouse brain homogenate) - see Swant data sheet.	In the mouse cerebellum, our immunostaining shows positive signal only in Purkinje cells (positive control) and no signal in other cell types (e.g. granule cells; negative control) in accordance with ample evidence in the literature, such as: Garcia-Segura et al. 1984; Rossi et al. 1993, 1995; Airaksinen et al. 1997; Buffo et al. 1997, 2000; Carulli et al. 2002; Carletti et al. 2008; Cupolillo et al. 2016; Higuera et al. 2017.
**anti-Cre**	Synaptic Systems, 257003	1:1500	Rabbit	By immunostaining on HEK293T cells transfected with a plasmid directing the expression of Cre (presence of staining) and on untransfected HEK293T cells (absence of staining) – Kang et al. 2023.<br>See also Ambrozkiewicz et al. 2017 and Lopez-Benito et al. 2018 for positive and negative controls using this antibody.	In the CNS, L7 promoter is known to be active only in Purkinje cells and in retinal bipolar neurons (Oberdick et al. 1990). In the cerebellum of AAV-PHP.eB-L7-Cre injected mice, several Cre^{+} Purkinje cells are detected (positive control), whereas no other cell types, such as granule cells (negative control), show Cre immunopositivity.
**anti-GFP**	Millipore, AB16901	1:1000	Chicken	By immunostaining on cells transfected with a plasmid directing the expression of GFP (presence of staining) and on cells that do not express GFP (absence of staining); by immunoblot (it recognises a single band of 30 kDa in lysates from E.coli expressing GFP but not in E.coli that do not express GFP) - see Millipore data sheet.	In the cerebellum of AAV-PHP.eB-L7-GFP injected mice, several GFP^{+} Purkinje cells are detected (positive control), whereas no other cell types, such as granule cells (negative control), show GFP immunopositivity.

**anti-Iba1**	Novus, NB 100-1028	1:300	Goat	By immunoblot (it recognises a single band of approx. 16 kDa in human frontal cortex, mouse brain and rat brain lysates and mouse lymph node lysates) – see Novus data sheet.	In the mouse brain, positive signal is detected in cells with microglia morphology and not in astrocytes or neurons, in accordance with ample evidence in the literature, such as: Ito et al. 1998; Ohsawa et al. 2000; Koning et al. 2009; Sosna et al. 2018; Parmigiani et al. 2022.
**anti-Iba1**	Abcam, ab178847	1:500	Rabbit	By immunoblot (it recognises a single band of approx. 16 kDa in mouse spleen whole cell lysate) – see Abcam data sheet.	In the mouse brain, positive signal is detected in cells with microglia morphology and not in astrocytes or neurons, in accordance with ample evidence in the literature, such as: Ito et al. 1998; Ohsawa et al. 2000; Koning et al. 2009; Sosna et al. 2018; Parmigiani et al. 2022.
**anti-NeuN**	Millipore, MAB377	1:500	Mouse	By western blot (it recognises three bands of 46-48 kDa in FGF-2-treated cultured bone marrow cells and in cultured rat cerebral cortical neurons) – see Jin et al. (2003) Exp Neurol.	In the mouse cerebellar cortex, our immunostaining shows positive signal in granule cells (positive control) and no signal in Purkinje cells (negative control), in accordance with evidence in the literature, such as: Mullen et al. 1992; Wolf et al. 1996; Weyer and Schilling, 2003.
**anti-Sema3A**	Santa Cruz, sc-1146	1:500	goat	By western blot and immunostaining on HEK293T cells transfected with a plasmid directing the expression of Sema3A (presence of staining) and on untransfected HEK293T (absence of staining) – Vo et al. 2013.	Positive and negative controls are reported in Vo et al. 2013.
**anti-TH**	Pelfreez, P40101-150	1:1000	Rabbit	By immunoblot (it recognises a single band of approx. 60 kDa in rat caudate lysate) – see Pel-Freez data sheet.	In the mouse midbrain, our immunostaining shows positive signal in cells in the substantia nigra pars compacta (positive control) and no signal in neurons in adjacent areas (negative control), in accordance with evidence in the literature, such as Thompson et al. 2009; Stott and Barker, 2014; Zhou et al. 2016.
**anti-TH**	Abcam, ab76442	1:500	Chicken	By immunoblot (it recognises a single band of approx. 58 kDa in mouse brain homogenate, which can be inhibited by the immunogen peptide) – see Abcam data sheet.	In the mouse midbrain, our immunostaining shows positive signal in cells in the substantia nigra pars compacta (positive control) and no signal in neurons in adjacent areas (negative control), in accordance with evidence in the literature, such as Thompson et al. 2009; Stott and Barker, 2014; Zhou et al. 2016.

Supplementary Table S1. Details of the primary antibodies used in this study.
